# Whole exome sequencing identifies a heterozygous missense variant in the *PRDM5* gene in a family with Axenfeld–Rieger syndrome

**DOI:** 10.1007/s10048-015-0462-0

**Published:** 2015-10-21

**Authors:** Shazia Micheal, Sorath Noorani Siddiqui, Saemah Nuzhat Zafar, Hanka Venselaar, Raheel Qamar, Muhammad Imran Khan, Anneke I. den Hollander

**Affiliations:** Department of Ophthalmology, Radboud University Medical Center, P.O. Box 9101, 6500 HB Nijmegen, The Netherlands; Department of Pediatric Ophthalmology, Al-Shifa Eye Trust Hospital Jhelum Road, Rawalpindi, Pakistan; Center for Molecular and Biomolecular Informatics, Radboud Institute for Molecular Life Sciences, Radboud University Medical Center, Nijmegen, The Netherlands; Department of Biosciences, COMSATS Institute of Information Technology, Islamabad, Pakistan; Al-Nafees Medical College and Hospital, Isra University, Islamabad, Pakistan; Department of Human Genetics, Radboud University Medical Center, Nijmegen, The Netherlands

**Keywords:** Axenfeld–Rieger syndrome, Whole exome sequencing, *PRDM5*

## Abstract

Axenfeld–Rieger syndrome (ARS) is a disorder affecting the anterior segment of the eye, often leading to secondary glaucoma and several systemic malformations. It is inherited in an autosomal dominant fashion that has been associated with genetic defects in *PITX2* and *FOXC1*. Known genes *CYP1b1*, *PITX2*, and *FOXC1* were excluded by Sanger sequencing. The purpose of current study is to identify the underlying genetic causes in ARS family by whole exome sequencing (WES). WES was performed for affected proband of family, and variants were prioritized based on in silico analyses. Segregation analysis of candidate variants was performed in family members. A novel heterozygous *PRDM5* missense variant (c.877A>G; p.Lys293Glu) was found to segregate with the disease in an autosomal dominant fashion. The novel missense variant was absent from population-matched controls, the Exome Variant Server, and an in-house exome variant database. The Lys293Glu variant is predicted to be pathogenic and affects a lysine residue that is conserved in different species. Variants in the *PRDM5* gene were previously identified in anterior segment defects, i.e., autosomal recessive brittle cornea syndrome and keratoconus. The results of this study suggest that genetic variants in *PRDM5* can lead to various syndromic and nonsyndromic disorders affecting the anterior segment of the eye.

## Introduction

Axenfeld–Rieger Syndrome (ARS; OMIM 180500) is a rare developmental disorder inherited in an autosomal dominant manner with an incidence of 1:200,000. ARS is a part of phenotypically heterogeneous group of conditions involving anterior segment dysgenesis, constituting a wide spectrum of developmental anomalies that may affect the cornea, iris, lens, and angle [1]. ARS is characterized by a broad range of abnormalities, with evident ocular and systemic manifestations.

Ocular features observed in ARS patients include iris stromal hypoplasia, polycoria, corectopia, iridogoniodysgenesis, posterior embryotoxon, and iris strands bridging the iridocorneal angle to the trabecular meshwork [2]. Increased ocular pressure (IOP) leading to glaucoma is the major consequence of the anterior segment dysgenesis observed in ARS, with approximately half of the patients developing secondary glaucoma [3]. In addition, ARS patients sometimes present with various systemic abnormalities including facial dysmorphisms (e.g., hypertelorism, telecanthus, maxillary hypoplasia with flattening of the mid-face, prominent forehead, and a broad, flat nasal bridge) and dental abnormalities (e.g., microdontia or hypodontia). In the abdominal region, a failure of involution of the skin results in redundant periumbilical skin. Hypospadia in males, anal stenosis, pituitary abnormalities, and growth retardation may also be observed. Systemic changes other than these are usually not considered as the classical features of ARS [4].

ARS has been associated with mutations in the *pituitary homeobox 2* (*PITX2*; OMIM 601542) gene at 4q25 [5], and the *forkhead box C1* (*FOXC1*; OMIM 601090) gene at 6p25 [6, 7]. A third locus was suggested on 13q14, but a disease-causing gene has not yet been identified [8, 9]. In two isolated ARS cases, deletion of the 16q23-q24 region [10] and deletion of the *PAX6* gene at 11p13 [11] have been reported.

The goal of this study was to identify the underlying genetic cause in an autosomal dominant ARS family by whole exome sequencing (WES).

## Methods

### Clinical evaluation

**Fig. 1:**
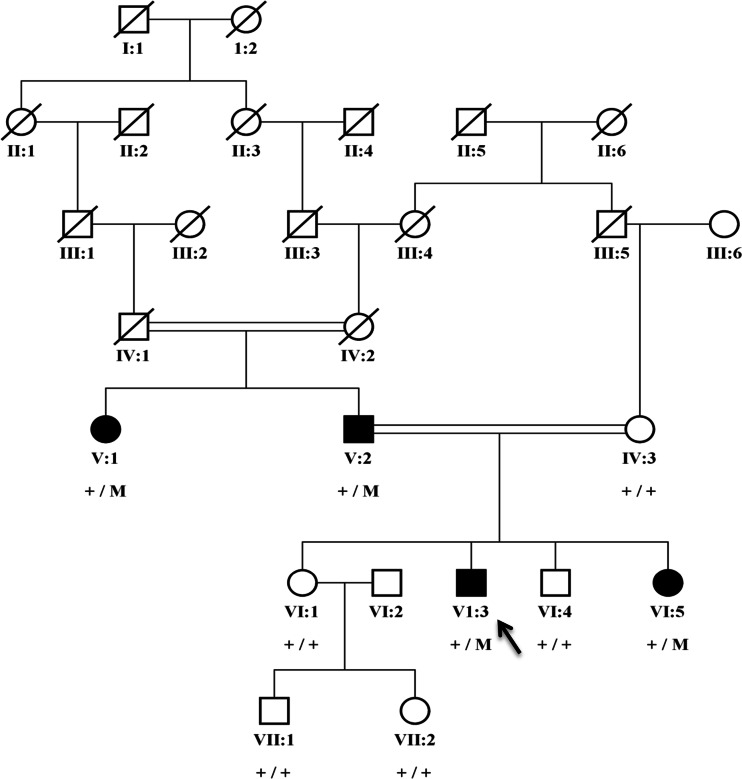
Segregation of a *PRDM5* missense variant in a family with Axenfeld–Rieger syndrome. The c.877A>G; p.Lys293Glu variant is indicated with an M, and the wild-type allele with a +. All affected individuals carry the variant heterozygously, while the unaffected individuals do not carry the variant. The proband is indicated with an *arrow*

A Pakistani family of Punjabi origin with four affected individuals was included in the study (Fig. 1). The study adhered to the principles of the declaration of Helsinki and was approved by the institutional ethical review board of Al-Shifa Eye Trust Hospital in Rawalpindi. Blood samples were drawn from affected and unaffected family members, after obtaining written informed consent. DNA was extracted using a standard phenol-chloroform method [12].

Clinical characterization of affected individuals included funduscopy, and measurement of intraocular pressure (IOP) with Goldmann applanation tonometry. Assessment of visual field defects was performed with a Humphrey Visual Field Analyzer (Carl Zeiss Humphrey Systems, Dublin, CA, USA). Anterior insertion of the iris into the trabecular meshwork with prominent iris processes was observed by gonioscopic examination. All participants were examined by a general physician for the presence or absence of systemic abnormalities.

### Sanger sequencing

Known genes *cyp1b1*, *Pitx2*, and *Foxc1* were excluded by direct Sanger sequencing of the coding exonic and flanking intronic regions of the respective genes. Conditions used to amplify these genes can be provided on request.

### Exome sequencing and analysis

To identify the underlying genetic cause of the disease in this family, WES was performed using genomic DNA of the proband (VI:3). Enrichment of exonic sequences was achieved by using the SureSelectXT Human All Exon V.2 Kit (50 Mb) (Agilent Technologies, Inc., Santa Clara, CA, USA). Sequencing was performed on a SOLiD 4 sequencing platform (Life Technologies, Carlsbad, CA, USA). Hg19 reference genome was aligned with the reads obtained using SOLiD LifeScope software V.2.1 (Life Technologies).

To evaluate the pathogenicity of the variants obtained from WES, bioinformatic analysis was performed using PhyloP (nucleotide conservation in various species), Grantham score (amino acid conservation), Sorting Intolerant from Tolerant (SIFT), MutationTaster, and PolyPhen2 (http://genetics.bwh.harvard.edu/pph2/). The presence of potential pathogenic variants was confirmed, and segregation was checked by PCR and Sanger sequencing. Sequencing was performed using the Big Dye Terminator Cycle Sequencing-Ready Reaction Kit (Applied Biosystems) on a 3130 DNA automated sequencer (Applied Biosystems, Foster City, CA, USA) using standard protocols.

### Protein conservation

PRDM5 protein sequences from different species were aligned to study the evolutionary conservation of the mutated amino acid Lys293 using Vector NTI Advance 2011.

### Protein structure prediction

Homology-based modeling using HOPE [13] was performed to assess the possible structural changes in the mutant protein. The human PDRM5 protein sequence (NM_018699, UniProt ID: Q9NQX1) was used to predict the wild-type and mutant protein structure.

## Results

### Clinical characterization

**Fig. 2:**
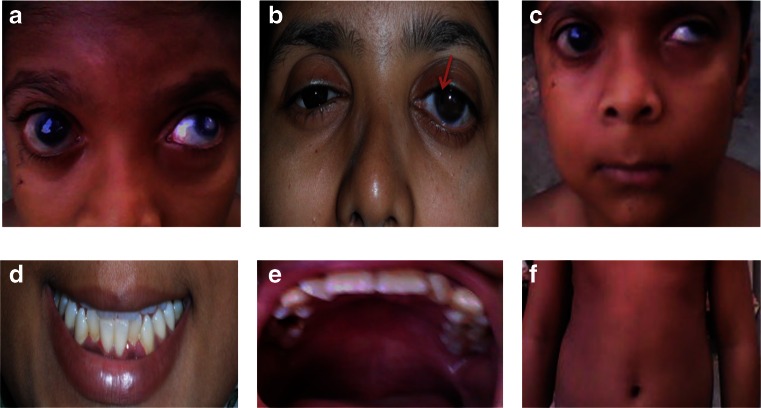
Ocular and systemic characteristics of the family with Axenfeld–Rieger syndrome. **a** Eyes of the proband (VI:3) presented bilateral buphthalmos, corectopia, polycoria, corneal edema, posterior embryotoxon, posterior subcapsular cataract, vascularized corneal opacity, and iris atropy patches in the left eye. **b** Both eyes of individual (VI:5) showed megalocornea and posterior embryotoxon. The right eye shows polycoria, and the left eye shows corectopia. **c** Facial dysmorphism in individuals VI:3 included flattening of the mid-face, a broad forehead, a broad nasal bridge, a thin upper lip with a long philtrum, a protruding lower lip, and a receding chin; **d** microdontia; **e** micrognathia; **f** redundant periumbilical skin

The proband (VI:3) has an affected father (V:2) and aunt (V:1), and one affected sister (VI:5) (Fig. 1). The proband was diagnosed at age 4 years. Ocular abnormalities included bilateral buphthalmos, correctopia, iris atrophy, corneal opacity, embryotoxon, posterior subcapsular cataract, and mild vitreous condensation. Funduscopy showed glaucomatous atrophy of the optic nerve with a cup-disc ratio (CDR) of 0.7 and 0.6 for the right and left eyes, respectively, and IOP measured by Goldmann applanation tonometry was 32 mmHg in the right eye and 36 mmHg for the left eye. The proband underwent several surgeries for the right eye, including trabeculectomy with mitomycin C (MMC) when he was 6 years old. For the left eye, cryopexy was done. At 7 years of age, cataract surgery was performed in the right eye, followed by parsplana viterectomy with silicone oil as he developed retinal detachment after trauma. He has typical facial features of ARS such as telecanthus, a broad nasal bridge, micrognathia, and microdontia. His abdominal features included redundant periumbilical skin. Facial dysmorphism in individuals VI:3 and VI:5 included flattening of the mid-face, a broad forehead, a broad nasal bridge, a thin upper lip with a long philtrum, a protruding lower lip and a receding chin (Fig. 2a–f). Both individuals V:1 and V:2 have hearing defects and hip joint anomalies. None of the affected individuals have cardiovascular defects.

Detailed clinical features of the proband and the other affected individuals are summarized in Table 1.

**Table 1:** Clinical evaluation of affected family members with Axenfeld–Rieger syndrome

Affected family members		V:1	V:2	VI:3	VI:5
Current age (years)		55	60	10	20
Gender		F	M	M	F
Eye					
	Iris dysplasia (goniodysgenesis)	+	+	+	+
	Iris hypoplasia	+	+	+	+
	Glaucoma	+	+	+	+
	Early development of nuclear cataract	−	−	+	−
	Polycoria	−	−	+	+
	Corectopia (displaced pupils)	+	+	+	+
	Megacornea	−	−	−	+
	Cataract	−	−	+	−
	Telecanthus	−	−	+	+
	Vitreous condensation	+	+	+	+
Ear					
	Abnormal ear, hearing defect	+	+	−	−
Nose					
	Broad nasal bridge	+	+	+	+
Teeth					
	Microdontia	−	+	+	−
	Micrognathia	+	+	+	−
Abdomen					
	Umbilical defect (redundant periumbilical skin)	+	+	−	+
Joints					
	Congenital hip anomalies	+	+	−	−

### WES variant selection and segregation analysis of the candidate variants identified

Variants obtained by WES analysis of the proband were prioritized based upon a dbSNP frequency <0.5, nucleotide and amino acid conservation, and by in silico analyses using online databases. No variants were present in the known ARS genes *PITX2* and *FOXC1*. Therefore, candidate variants present in known genes involved in autosomal dominant anterior segment defects and glaucoma [1] (Table 2) were screened for segregation in the family.

**Table 2:** In silico and segregation analysis of variants present in genes involved in anterior segment defects and glaucoma

Gene name	cDNA change	Amino acid change	Phylo P	Grantham distance	SIFT	Polyphen-2	MutationTaster	Segregation
*CYP1B1*	c.1103G>A	p.Arg368His	5.53	29	Deleterious	Probably damaging	Disease-causing	No
*MYOC*	c.227G>A	p.Arg76Lys	0.45	26	Tolerated	Benign	Polymorphism	No
*OPTN*	c.964G>C	p.Glu322Gln	2.79	29	Deleterious	Benign	Polymorphism	No
*COL4A1*	c.1673C>T	p.Ala558Val	3.03	64	Tolerated	Benign	Disease-causing	No
*WDR36*	c.790A>G	p.Ile264Val	1.01	29	Tolerated	Benign	Polymorphism	No
***PRDM5***	**c.877A>G**	**p.Lys293Glu**	**2.71**	**56**	**Deleterious**	**Benign**	**Disease-causing**	**Yes**
*B3GALTL*	c.1108G>A	p.Glu370Lys	2.30	56	Deleterious	Probably damaging	Polymorphism	No

The variant in *PRDM5* segregating with disease is shown in bold letters

**Fig. 3:**
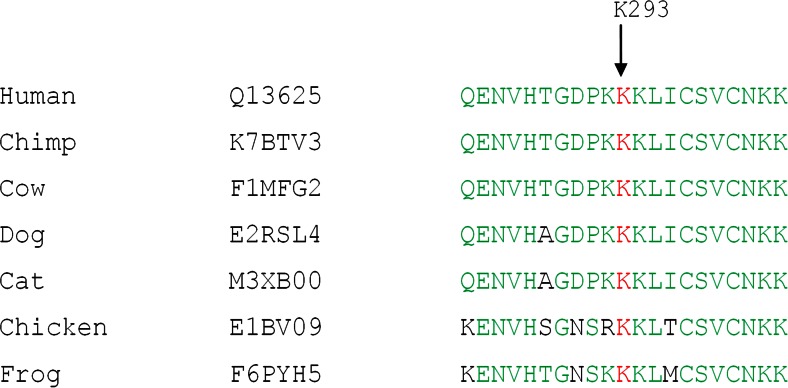
PRDM5 protein sequence alignment (amino acids 283–303) across species, indicating evolutionary conservation of lysine at position 293 in human PRDM5

A novel, heterozygous missense variant (c.877A>G; p.Lys293Glu) in the *PRDM5* gene was found to segregate with the disease in the family (Fig. 1). The variant (c.877A>G; p.Lys293Glu) was absent in population matched controls and is also absent in the Exome Variant Server database (http://eversusgs.washington.edu/EVS/) and in the 1000 Genomes Project (http://browser.1000genomes.org/index.html). The variant was predicted to be pathogenic by most in silico analyses and is present in a Cys_2_His_2_ zinc finger protein domain of PRDM5. The lysine residue affected by the variant (c.877A>G; p.Lys293Glu) is conserved in all species analyzed (Fig. 3).

### Protein structure prediction

**Fig. 4:**
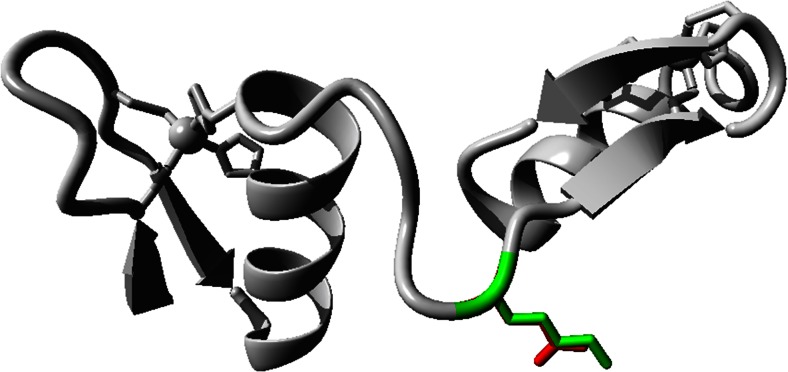
Secondary structure of the PRDM5 protein. The Lys residue in the linker region between two Zn-finger domains is represented with the *green color*, and the mutant Glu variant is indicated with a *red color*

Hope project analysis predicted that the lysine residue at position Lys293 is located in the linker between two Zn-finger domains (Fig. 4). The side chain seems to be completely exposed to the exterior surface. The lysine residue is large in size, is basic, and is positively charged, while the mutant glutamic acid (Glu) is smaller in size, is acidic, and is negatively charged. The wild-type Lys residue might have been involved in electrostatic interactions with other domains of the same protein, or with other molecules in a complex. The mutation introduces a residue with a sidechain that is smaller and that carries an opposite charge, and therefore, the interactions may be lost.

## Discussion

In the current study, WES identified a novel pathogenic missense variant (c.877A>G; p.Lys293Glu) in the *PRDM5* gene segregating with the disease in a family with autosomal dominant ARS. Previously, variants in the *PRDM5* gene have been identified in autosomal recessive brittle cornea syndrome (BCS), and an enrichment of potentially pathogenic heterozygous variants has been observed in keratoconus [4, 14]. In BCS patients carrying *PRDM5* mutations, a relationship was noted between the zygosity of the mutation (homozygous/heterozygous) and the clinical features of the patients and age of onset of keratoconus. Heterozygous carriers of *PRDM5* mutations have mildly reduced central corneal thickness, mild keratoconus, and blue sclera compared to individuals with homozygous mutations, who have more severe features. Individuals with BCS who carry a homozygous deletion of exons 9–14 have an earlier age of onset and more severe keratoconus compared to individuals carrying a mutation heterozygously [4]. A recent study proposed that heterozygous variants in *PRDM5* and *ZNF469* predisposed the patients toward the development of isolated keratoconus [15]. These studies support that a range of ocular phenotypes are associated with variants in the *PRDM5* gene, and the current study extends the spectrum to autosomal dominant ARS.

*PRDM5* localizes to human chromosome 4q26 and encodes a member of the family of PRDM proteins [16]. PRDM proteins constitute a family of transcription regulators characterized by the presence of variable number of zinc finger repeats typically involved in protein–DNA or protein–protein interaction [17], and an N-terminal PR domain which shares similarity to the SET domain of histone methyltransferases. PRDM proteins play a vital role in gene expression regulation, either directly or indirectly by modifying the structure of chromatin, i.e., through the intrinsic methyltransferase activity, or via the recruitment of chromatin remodeling complexes, respectively. PRDM proteins typically display tissue-specific patterns of expression and are often involved in the differentiation of specific cell lineages [18].

PRDM5 is involved in the development and maintenance of the extracellular matrix (ECM), which explains its involvement in BCS and ARS syndromes, which represent multisystemic connective tissue disorders. Quantitative PCR analysis of fibroblast RNA from BCS patients and control individuals identified significant differences in genes involved in the ECM. In particular, genes encoding fibrillar collagens (e.g., *COL4A1* and *COL11A1*), connective tissue components (e.g., *HAPLN1*), and molecules regulating cell migration and adhesion (e.g., *EDIL3* and *TGFB2*) were significantly downregulated. *EDIL3*, *HAPLN1*, and *COL11A1* each demonstrated a greater than 30-fold decrease in mutant lines relative to controls [4].

A phenotypic overlap is also present to some extent in BCS and ARS. For example, in BCS, sensorineural hearing loss is a prominent feature and was observed in families with mutations in *PRDM5* described previously by Burkitt Wright et al. 2011 [4]. In the current study, patients V:1 and V:2 with ARS, a progressive sensorineural hearing loss was apparent at young age and became severe in adulthood, especially in individual V:2. In addition to this, most of the affected individuals with BCS described previously had experienced hip problems, similar to the patients V:1 and V:2 with ARS in current study. The central corneal thickness is reduced in patients with BCS, which is also reported previously in patients with ARS who underwent a corneal transplantation and carry mutations in *PITX2* gene [19].

In zebrafish, *Prdm5* expression was observed in specific tissues including intestinal mucosa, ventral spinal cord and ciliary zone by in situ hybridization [16]. In addition, *Prdm5* is highly expressed in the osteoblast region of developing bones in mice [20]. Loss of *Prdm5* results in delayed ossification, involving a prominent impairment in the assembly of fibrillar collagens. The presence of Prdm5 is therefore vital for the proper assembly of the osteoblastic extracellular matrix [20].

To study the role of Prdm5 during zebrafish development, both loss of function and gain of function approaches have been used. Two morpholino oligonucleotides were designed to obtain a depletion of Prdm5 protein: one targeting the region comprising the start codon (ATGmo), and one targeting the exon1/intron1 splice site (splice blocking or SBmo). The ATGmo induced cyclopia, while the SBmo induced closer, smaller eyes and marked axial mesendodermal defects in the jaw, heart, and blood [16]. This is in line with the eye, jaw, and heart defects and hip anomalies seen in individuals affected by ARS.

Other genes involved in ARS, *FOXC1* [1, 6, 7], and *PITX2* [5] also encode transcription factors. In addition, mutations in transcription factor *PITX3* have been identified in anterior segment dysgenesis and congenital cataracts [5, 21]. These studies, together with the results presented here, support an important role for transcription factors and regulators in the development of anterior segment defects.

Notably, nearly all homozygous *PRDM5* mutations detected in BCS patients disrupt the open reading frame, suggesting that BCS is associated with loss of function of PRDM5 [4]. In this study, a heterozygous missense variant in the linker region between two zinc finger domains of PRDM5 was identified in autosomal dominant ARS. Although the exact mechanism of action of this variant is unknown, it might have a dominant negative effect, leading to distinct ocular and extraocular features than observed in BCS. Mutations in the *PITX2* gene are associated with autosomal dominant ARS, but both autosomal dominant and recessive mutations have been identified in another anterior segment defect (i.e., ring dermoid of the cornea) [22]. Both PRDM5 and PITX2 are involved in Wnt signaling pathways and are involved in the development of multiple organs [16, 23]. This suggests that *PRDM5* and *PITX2* mutations can lead to a spectrum of syndromic and nonsyndromic anterior segment defects, with autosomal dominant and recessive inheritance patterns. In summary, we describe a novel association of a *PRDM5* missense variant with autosomal dominant ARS.
